# Males Receive Low-Tidal Volume Component of Lung Protective Ventilation More Frequently than Females in the Emergency Department

**DOI:** 10.5811/westjem.2020.2.45191

**Published:** 2020-04-16

**Authors:** Derek L. Isenberg, Benjamin Bloom, Nina Gentile, Hannah Reimer, Owen D. Glaze, Paige Palumbo, Rachel Fenstermacher

**Affiliations:** Lewis Katz School of Medicine, Department of Emergency Medicine, Philadelphia, Pennsylvania

## Abstract

**Introduction:**

Mechanical ventilation is a commonly performed procedure in the emergency department (ED). Approximately 240,000 patients per year receive mechanical ventilation in the ED representing 0.23% of ED visits. An ED-based trial published in 2017 showed that a bundle of interventions in mechanically ventilated patients, including low tidal volume ventilation, reduced the development of acute respiratory distress syndrome by nearly 50%. Prior literature has shown that as many as 40% of ED patients do not receive lung protective ventilation. Our goal was to determine whether differences exist between the percent of males vs females who are ventilated at ≥ 8 milliliters per kilogram (mL/kg) of predicted body weight.

**Methods:**

We conducted this study at Temple University Hospital, a tertiary care center located in Philadelphia, Pennsylvania. This was a planned subgroup analysis of study looking at interventions to improve adherence to recommended tidal volume settings. We used a convenience sample of mechanically ventilated patients in our ED between September 1, 2017, and September 30, 2018. All adult patient > 18 years old were eligible for inclusion in the study. Our primary outcome measure was the number of patients who had initial tidal volumes set at > 8 mL/kg of predicted body weight. Our secondary outcome was the number of patients who had tidal volumes set at ≥ 8 mL/kg at 60 minutes after initiation of mechanical ventilation.

**Results:**

A total of 130 patients were included in the final analysis. We found that significantly more females were initially ventilated with tidal volumes ≥ 8 mL/kg compared to men: 56% of females vs 9% of males (p=<0.001). Data was available for 107 patients (82%) who were in the ED at 60 minutes after initiation of mechanical ventilation. Again, a significantly larger percentage of females were ventilated with tidal volumes ≥ 8 mL/kg at 60 minutes: 56% of females vs 10% of males (p<0.001).

**Conclusion:**

The vast majority of tidal volumes ≥ 8 mL/kg during mechanical ventilation occurs in females. We suggest that objective measurements, such as a tape measure and tidal volume card, be used when setting tidal volumes for all patients, especially females.

## INTRODUCTION

Mechanical ventilation is a commonly performed procedure in the emergency department (ED). Approximately 240,000 patients per year receive mechanical ventilation in the ED, representing 0.23% of ED visits.^1^ The landmark LOV-ED trial published in 2017 showed that a bundle of interventions in the ED for mechanically ventilated patients reduced the development of acute respiratory distress syndrome (ARDS) and related complications by nearly 50%.^2^ One item in the bundle was low tidal volume ventilation defined as 6–8 milliliters per kilogram (mL/kg) of predicted body weight (PBW). An earlier 2012 meta-analysis by Neto et al showed that an early low tidal volume ventilation strategy in patients without ARDS/acute lung injury resulted in lower rates of development of ARDS, decreased rates of pulmonary infection, and decreased mortality rates.^3^ Prior literature has shown that as many as 40% of ED patients do not receive lung protective ventilation.^4^ Risks of ventilating patients with larger tidal volume include barotrauma, pneumothorax, and damage to surfactant in the lungs.^5^,^6^

Our goal was to determine whether differences exist between the percent of males vs females who are ventilated at ≥ 8mL/kg of PBW at the time of initial ventilator settings and at 60 minutes after the initiation of mechanical ventilation.

## METHODS

This study took place at Temple University Hospital (TUH), a tertiary care center located in Philadelphia, Pennsylvania. TUH is a 599-bed tertiary care center with 84,433 annual ED visits. TUH has a postgraduate year (PGY) 1–3 emergency medicine (EM) residency with 38 residents. The majority of intubations are performed by the PGY-2 and PGY-3 residents in the ED.

This was a planned subgroup analysis of study looking at interventions to improve adherence to recommended tidal volume settings. This study used a convenience sample of mechanically ventilated patients in our ED between September 1, 2017 – September 30, 2018. Our ED has research associates (RA) in the department for 12 hours per day, seven days a week during academic semesters. These RAs are responsible for screening patients for various ED-based studies as well as recording study data in real time. Mechanically ventilated patients were only enrolled when the RAs were physically in the department to collect data.

All adult patient ≥ 18 years old were eligible for inclusion in the study. Exclusion criteria included patients younger than 18 years of age, prisoners, pregnant women, and trauma patients (as the ventilators are set by the trauma surgery service rather than the emergency physicians). Patients who were intubated by or had their intubations supervised by one of the investigators were also excluded.

In our ED, emergency physicians (faculty and residents) are responsible for setting the tidal volumes for mechanically ventilated patients. Nurses will enter a patient’s height and weight into the electronic health record, but these values are typically estimated rather than measured. Physicians had access to a measuring tape and tidal volume chart but were not required to measure the patients’ heights. After the ventilator had been set and routine, post-intubation care was completed, one of the RAs measured the patient with the tape measure and calculated the PBW of the patient. PBW was calculated using the same formulas that were used in prior ARDS studies.^7^,^8^ All emergency physicians were blinded as to the hypothesis and purpose of this study.

**Population Health Research Capsule**

What do we already know about this issue?

Lung protective ventilation reduces complications from acute respiratory distress syndrome, but it is infrequently practiced in the emergency department (ED).

What was the research question?

Do male and female patients receive lung protective ventilation at the same frequency in the ED?

What was the major finding of the study?

Males received initial lung protective ventilation at significantly higher rates than females: 91% vs 44%.

How does this improve population health?

Objective measurements, such as a tape measure and tidal volume card, should be used when setting ventilators for all patients, especially females.

Our primary outcome measure was the number of patients stratified by gender who had initial tidal volumes set ≥8 mL/kg of PBW. Our secondary outcome was the number of patients stratified by gender who had tidal volumes set ≥8 mL/kg by 60 minutes post-intubation. We hypothesized tidal volume settings might be titrated within the first hour of mechanical ventilation. In our ED, all ventilator changes within the first hour are made by the treating emergency physicians.

We performed data analysis using descriptive statistics and compared the groups using the chi-squared method to determine p values. This study was approved by the institutional review board at Temple University.

## RESULTS

We included 130 patients in the final analysis, and 107 patients had data available at 60 minutes. Twenty-three patients left the ED prior to one hour, either admitted to an intensive care unit or operating room; 38% of the patients were female. The mean age was 57 years with a range of 21–89 years. The patients’ demographic information is listed in Table 1.

We found that significantly more females were initially ventilated at tidal volumes ≥ 8mL/kg compared to men: 56% of females vs 9% of males (p=<0.001) (Table 2). Data was available for 107 patients (82%) who were still in the ED at 60 minutes after initiation of mechanical ventilation. Again, a significantly larger percentage of females were ventilated at tidal volumes ≥ 8mL/kg at 60 minutes: 56% of females vs 10% of males (p<0.001).

## DISCUSSION

In the analysis of our data, we found that females were more frequently ventilated at tidal volumes ≥ 8mL/kg compared to males. This has been reported before in intensive care and operating room literature, but not in the EM literature. Han et al found, in their multivariate analysis, that the high tidal volumes for women during mechanical ventilation were related to their height rather than their gender.^9^ Lellouche et al studied mechanical ventilation in patients after cardiac surgery.^10^ His group found that women and obese patients (≥ 30kg per meter squared) were less likely to receive lung protective ventilation.

We suspect that clinicians may not appreciate the difference in PBW between males and females of the same height. (Calculations available at http://www.ardsnet.org/files/pbwtables_2005-02-02.pdf). For example, at 152 centimeters (cm) (60 inches), 8 mL/kg of PBW is 368 mL for female and 400 mL for men. At 168 cm (66 inches), 8 mL/kg of PBW is 475 ml for females and 512 mL for males. It is also possible that physicians do not feel comfortable setting a ventilator at or below 400 mL. Because of the discrepancy between the tidal volumes between males and females, clinicians should be encouraged to use an objective measure of PBW when setting a ventilator.

## LIMITATIONS

This study focused on ventilator settings and did not evaluate mortality or duration of mechanical ventilation. It is possible that tidal volumes over 8 mL/kg did not adversely affect patient-oriented outcomes. In addition, this was a convenience sample of patients and may not reflect the entirety of patients who received mechanical ventilation in the ED. Finally, this is a single, tertiary care center and thus results may not apply to all centers.

## CONCLUSION

The vast majority of patients with tidal volumes ≥ 8mL/kg of PBW during mechanical ventilation are females. This difference did not improve after 60 minutes, although clinicians had time to adjust the ventilator. We suggest that objective measurements, such as a tape measure and tidal volume card, be used when setting ventilators for all patients, especially females.

## Figures and Tables

**Table 1:** Demographics of patients in study examining lung protective ventilation by gender in the emergency department.

	Men, n (%)	Women, n (%)	P-value
Patients initially ventilated at tidal volumes ≥ 8mL/kg	7 (9)	28 (56)	p<0.001
Patients ventilated at tidal volumes ≥ 8mL/kg at 60 minutes	6 (10)	23 (56)	p<0.0001

*ml/kg*, milliliters per kilogram.

**Table 2:** Results of study comparing tidal volume settings by gender.

Variables	Male	Female	All patients
Age, years (range)	59.0 (21–87)	62.6 (26–98)	57.3 (21–98)
Mean height, cm (range)	175.5 (143–194)	161.4 (145–175)	170.1 (143–191)
Number of patients with initial ventilator settings, n (%)	80 (62)	50 (38)	130 (100)
Number of patients with ventilator settings at 60 minutes, n (%)	62 (58)	45 (43)	107 (82)
Mean initial tidal volume, mL/kg PBW (range)	6.2 (5.3–9.1)	8.2 (7.4–9.3)	
Mean initial tidal volume, mL (range)	481 (375–600)	440 (300–550)	465 (300–600)
Mean tidal volume at 60 minutes, mL(range)	477 (375–600)	447 (350–550)	504 (375–600)
Indications for mechanical ventilation, n (%)			
Respiratory failure	15 (19)	25 (50)	40 (31)
Cardiac arrest	20 (25)	6 (12)	26 (20)
Altered mental status/Overdose	21 (26)	9 (18)	30 (23)
Seizure/Status epilepticus	10 (13)	3 (6)	13 (10)
Angioedema/Airway infection	5 (6)	4 (8)	9 (8)
Stroke	4 (5)	2 (4)	6 (5)
Gastrointestinal bleeding	2 (3)	0 (0)	2 (1)
DKA	1 (1)	1 (2)	2 (1)
Burns/Smoke inhalation	2 (3)	0 (0)	2 (1)

*Cm*, centimeters; *ml/kg*, milliliters per kilogram; *PBW*, predicted body weight; *DKA*, diabetic ketoacidosis.
